# B-cell receptor repertoire remodeling in Kawasaki disease is associated with clonal expansion and distinct repertoire features in coronary artery lesions

**DOI:** 10.3389/fimmu.2026.1994376

**Published:** 2026-10-14

**Authors:** Yuanyuan Xu, Xixiong Lin, Yanfei Chen, Xing Zhang, Long Ma, Xinsheng Yao, Jun Li

**Affiliations:** 1 Department of Immunology, Center of Immunomolecular Engineering, Zunyi Medical University, Zunyi, Guizhou, China; 2 Department of Cardiology, the Affiliated Children’s Hospital of Kunming Medical University, Kunming, Yunnan, China

**Keywords:** B-cell receptor repertoire, coronary artery lesions, IgA, IgM, Kawasaki disease

## Abstract

**Background:**

Kawasaki disease (KD) is an acute systemic vasculitis in children, but the characteristics of the B-cell receptor (BCR) repertoire during acute disease and their association with coronary artery lesions (CALs) remain poorly understood.

**Methods:**

High-throughput sequencing of BCR heavy-chain (BCR-H) CDR3 regions was performed in 22 children, including 9 healthy controls and 13 patients with KD, of whom 9 had no CAL and 4 had CAL. Repertoire diversity, clonal expansion, immunoglobulin isotype distribution, V–J gene usage, CDR3 characteristics, and inter-individual and cross-isotype repertoire sharing were systematically evaluated.

**Results:**

KD exhibited reduced BCR repertoire diversity, with a significant decrease in IgM diversity. The repertoire showed increased contributions of high-frequency and hyperexpanded clonotypes, with the most pronounced expansion in patients with CAL. IgM displayed the strongest clonal remodeling, characterized by reduced low-frequency and increased highly abundant clonotypes. KD also showed selective alterations in IGHV usage and V–J gene pairing. CAL was associated with increased IGHA usage and changes in CDR3 amino acid composition. Substantial inter-individual CDR3 sharing was observed among KD patients, and 20 recurrent clonotypes absent from healthy controls were identified as candidate KD-associated sequences. Cross-isotype CDR3 sharing also tended to be higher in KD, particularly among patients with CAL.

**Conclusions:**

Acute KD is characterized by substantial BCR repertoire remodeling, marked by selective IgM clonal expansion, repertoire convergence, and CAL-associated IgA-biased remodeling. These findings provide new insights into humoral immune dysregulation in KD and identify candidate B-cell clonotypes for further investigation of disease pathogenesis and coronary involvement.

## Introduction

Kawasaki disease (KD) is an acute systemic vasculitis that predominantly affects young children and remains a major cause of acquired cardiovascular disease in childhood (1). Although the etiology of KD remains incompletely understood, accumulating evidence supports a multifactorial process involving genetic susceptibility, environmental or infectious triggers, and dysregulated innate and adaptive immune responses (2, 3). During the acute phase, extensive activation of circulating leukocytes and inflammatory signaling pathways contributes to endothelial injury and vascular inflammation, while persistent inflammation and tissue infiltration are closely associated with coronary artery involvement (4, 5). Coronary artery lesions (CALs), including coronary artery dilatation and aneurysms, represent the most important complication of KD and determine a substantial proportion of its long-term cardiovascular burden (1, 6, 7). The management of KD and prevention of coronary complications also impose substantial healthcare and economic burdens (8). Recent transcriptomic and single-cell studies have further demonstrated that patients with CAL exhibit distinct immune activation patterns, including alterations in immunoglobulin-related and adaptive immune programs, suggesting that adaptive immunity may contribute to the transition from systemic inflammation to coronary vascular injury (9). However, the molecular characteristics of adaptive immune responses associated with CAL remain incompletely defined.

B cells and antibody-mediated immunity are increasingly recognized as important components of KD immunopathology. Early histopathological studies demonstrated abundant IgA-producing plasma cells within vascular tissues, including coronary arteries, during acute KD, providing evidence for a prominent local humoral immune response (10). Subsequent studies further demonstrated oligoclonal IgA responses in affected tissues and suggested that these antibodies may recognize antigens derived from infectious or environmental exposures (11). Consistent with these observations, circulating IgA levels have been associated with CAL development and coronary aneurysm severity, supporting a potential relationship between IgA-associated humoral responses and coronary vascular injury (12). More broadly, B-cell activation involves antigen-dependent selection and clonal expansion, followed by processes such as somatic hypermutation, affinity maturation, and immunoglobulin class switching that shape the antibody repertoire (13).

Recent studies have begun to characterize adaptive immune repertoire abnormalities in KD. An earlier immune-repertoire analysis identified reduced B-cell diversity, disease-associated V(D)J usage patterns, and distinct complementarity-determining region 3 (CDR3) amino acid features in KD, suggesting selective B-cell responses during acute disease (14). More recently, comprehensive BCR and TCR repertoire profiling demonstrated oligoclonal expansion and reduced diversity in the IgA and T-cell receptor beta (TCR-β) chain repertoires of patients with KD, further supporting the presence of antigen-associated adaptive immune selection (15). In parallel, single-cell transcriptomic analyses have identified altered B-cell developmental trajectories and increased immunoglobulin- and adaptive-immunity-associated programs in patients with CAL, providing additional evidence for B-cell involvement in coronary complications (9). Nevertheless, several important aspects of the KD BCR repertoire remain insufficiently characterized. In particular, whether acute KD preferentially remodels specific immunoglobulin isotypes, how clonal expansion is distributed across IgM, IgG, and IgA repertoires, and whether coronary involvement is associated with distinct V–J gene pairing or CDR3 sequence features remain unclear. Furthermore, the extent to which KD patients share recurrent BCR-H CDR3 sequences and whether identical clonotypes occur across different isotype compartments have not been systematically investigated. These questions are particularly relevant because recurrent or convergent BCR sequences may reflect common antigenic selection, whereas cross-isotype sharing may provide evidence of related clonal responses during B-cell differentiation (16, 17).

Therefore, to address these gaps, this study aimed to comprehensively characterize BCR heavy-chain CDR3 repertoire remodeling in children with acute KD and healthy controls. RNA isolated from peripheral blood samples of children with acute KD and healthy controls was subjected to high-throughput sequencing of BCR heavy-chain CDR3 regions to comprehensively characterize repertoire diversity, clonal architecture, immunoglobulin isotype distribution, V–J gene usage, CDR3 sequence characteristics, and inter-individual repertoire sharing. Particular attention was given to IgM-associated clonal remodeling, CAL-associated repertoire features, recurrent KD-associated clonotypes, and cross-isotype sharing. By integrating these complementary repertoire features, we sought to define the major characteristics of BCR remodeling during acute KD and to explore their potential relationship with coronary artery involvement.

## Materials and methods

### Study participants and sample collection

This study was approved by the Ethics Committee of Kunming Children’s Hospital (approval No. 2022-03-089-K-01), and written informed consent was obtained from the parents or legal guardians of all participants. Children diagnosed with KD, including those with coronary artery lesions (CAL), and age-matched healthy children undergoing routine physical examinations at Kunming Children’s Hospital between 2022 and 2024 were enrolled. A total of 22 children were included in the study, comprising 9 healthy controls (NC), 13 patients with KD, including 9 patients without CAL (no-CAL) and 4 patients with CAL. Healthy controls were children without a history of KD or other acute or chronic infectious, inflammatory, autoimmune, cardiovascular, hematological, or other major systemic diseases, and were considered clinically healthy based on routine physical examination and medical history. Peripheral blood samples were collected during the acute phase of KD and before intravenous immunoglobulin (IVIG) treatment. For each participant, peripheral blood samples were collected for BCR repertoire analysis. Clinical characteristics and sample grouping information are provided in **Supplementary Table 1**.

### Inclusion and exclusion criteria

Patients were eligible for the KD group if they (1) fulfilled the diagnostic criteria of the 2017 American Heart Association Scientific Statement on the diagnosis, treatment, and long-term management of KD; (2) were newly diagnosed and hospitalized during the acute phase of KD; (3) had complete demographic information, medical history, and echocardiographic data; and (4) underwent echocardiographic examination before discharge.

Patients were excluded if they had congenital heart disease, congenital or acquired valvular heart disease, acute or chronic myocardial infarction, arrhythmia, cardiomyopathy, pulmonary hypertension, hypertensive heart disease, other infectious, autoimmune, exanthematous, or hematological diseases, gastrointestinal disorders, or previous treatment with medications that could affect hematological parameters. Patients presenting during the subacute or convalescent phase of KD were also excluded.

### Definition of coronary artery lesions

KD patients were further classified according to the presence of CALs based on the 2020 Japanese Circulation Society/Japanese Society for Cardiovascular Surgery guidelines for the diagnosis and management of cardiovascular sequelae of KD. Briefly, coronary artery dilatation was defined as a coronary artery diameter ≥2.5 mm in children aged ≤3 years, ≥3.0 mm in children aged >3 to 9 years, and ≥3.5 mm in children aged >9 to 14 years. A coronary artery diameter of 4–7 mm was defined as a coronary artery aneurysm (CAA), whereas a diameter ≥8 mm was defined as a giant CAA. According to these criteria, the KD cohort comprised 9 patients without CAL and 4 patients with CAL.

### Primer design and synthesis

To amplify BCR-H CDR3 regions, seven upstream primers targeting the human IGHV gene families (IGHV1–IGHV7) and three downstream primers targeting the human immunoglobulin constant regions IGHA, IGHG, and IGHM were designed and synthesized. All primers were synthesized by Invitrogen (Thermo Fisher Scientific). The primer sequences used for BCR-H CDR3 amplification are provided in **Supplementary Table 2**.

### Peripheral blood collection and PBMC isolation

Peripheral blood was collected from each participant into two tubes (approximately 2 mL in total). Blood samples were centrifuged at 400 × g for 20 min. Plasma was collected and stored in sterile tubes, while the leukocyte-containing layer was transferred to a 15 mL centrifuge tube for subsequent processing. Peripheral blood mononuclear cells (PBMCs) were isolated using a standard density-gradient centrifugation procedure and subsequently stored at −80 °C until RNA extraction.

### PBMC RNA extraction and reverse transcription

Total RNA was extracted from PBMCs using a standard RNA extraction procedure according to the manufacturer’s instructions. RNA concentration and quality were assessed before downstream library preparation. For each sample, the extracted RNA was used as the template for first-strand cDNA synthesis. Reverse transcription was performed using the RevertAid First Strand cDNA Synthesis Kit according to the manufacturer’s protocol.

### BCR-H CDR3 library preparation and high-throughput sequencing

BCR-H CDR3 libraries were generated using a multiplex polymerase chain reaction (PCR)-based strategy. Briefly, cDNA from individual PBMC samples was amplified using the IGHV family-specific upstream primer mixture together with downstream primers specific for IGHA, IGHG, or IGHM. The resulting amplicons containing the BCR-H CDR3 region were subjected to subsequent purification and library preparation according to the manufacturer’s protocol.

The amplified products were separated and purified by gel extraction. Purified target fragments were subjected to quality control before high-throughput sequencing. Samples that passed quality assessment were submitted to BGI Genomics for sequencing. Libraries meeting the quality-control criteria were sequenced using the DNBSEQ platform. Sequencing generated high-throughput BCR-H CDR3 repertoire data for each participant.

### BCR-H CDR3 repertoire processing and clonotype assignment

Raw sequencing data were processed using MiXCR for V(D)J alignment, CDR3 identification, and clonotype assembly. The resulting productive BCR-H CDR3 sequences were used for downstream repertoire analysis.

To obtain high-confidence BCR-H CDR3 sequences, the MiXCR output tables were subjected to additional filtering. Sequences were retained only when the amino acid sequence in the aaSeqCDR3 column started with cysteine (C) and ended with tryptophan (W). Sequences for which the allCHitsWithScore annotation was not assigned to IGHA, IGHG, or IGHM were excluded. The resulting filtered sequences were used for subsequent BCR repertoire analyses.

A clonotype was defined based on a unique nucleotide sequence, such that identical nucleotide sequences were considered the same clonotype. For each sample, the numbers of productive BCR-H CDR3 sequences and unique clonotypes were calculated. In addition to analyses of the overall BCR-H repertoire, BCR-H repertoires were stratified according to immunoglobulin isotype (IgA, IgG, and IgM) based on the constant-region annotation.

### BCR repertoire diversity and clonality analysis

BCR-H repertoire diversity was evaluated using the inverse Simpson diversity index. Higher inverse Simpson values indicate greater repertoire diversity, whereas lower values indicate reduced diversity. To characterize the clonal architecture of the BCR repertoire, clonotypes were ranked according to their clone counts. Highly abundant clonotypes were evaluated according to their rank distribution, including the top 1–10, 11–100, 101–1,000, 1,001–3,000, and 3,001–1,000,000 clonotypes. Clonotypes with clone counts ≤100 were defined as rare clonotypes and further categorized according to clone count as 1, 2–3, 4–10, 11–30, 31–100, and 101–maximum.

To further characterize the distribution of clonotypes across the repertoire, clonotypes were classified according to their relative abundance into four frequency categories: low-frequency (0–0.0001), intermediate-frequency (0.0001–0.001), high-frequency (0.001–0.01), and hyperexpanded (0.01–1). The cumulative proportion of clonotypes within each frequency interval was calculated to assess the clonal structure and distribution of the BCR repertoire. These analyses were performed for the total BCR-H repertoire and separately for IgA, IgG, and IgM repertoires.

### IGHV, IGHJ, and constant-region gene usage analysis

IGHV and IGHJ gene usage frequencies were calculated based on productive BCR-H CDR3 sequences. The relative usage of individual IGHV and IGHJ genes was compared between KD and healthy control groups. IGHV–IGHJ pairing frequencies were additionally calculated to identify preferential V–J gene combinations and disease-associated alterations in V–J usage.

Constant-region usage was assessed according to the IGHA, IGHG, and IGHM annotations. The relative contributions of IgA-, IgG-, and IgM-associated BCR-H CDR3 sequences were compared between KD and healthy controls and further examined according to CAL status.

### BCR-H CDR3 length and amino acid usage analysis

The amino acid length of each BCR-H CDR3 sequence was calculated, and the distribution and mean CDR3 length were compared between study groups. Amino acid usage frequencies within BCR-H CDR3 sequences were also calculated to characterize compositional differences between KD and healthy controls. Additional analyses were performed after stratification of KD patients according to the presence or absence of CALs.

### BCR repertoire overlap and shared clonotype analysis

To assess repertoire convergence between individuals, shared BCR-H CDR3 sequences were identified based on identical CDR3 sequences. The number of shared clonotypes among samples and groups was calculated. Pairwise repertoire similarity was further quantified using the Jaccard index, defined as the number of shared clonotypes divided by the total number of unique clonotypes present in the compared repertoires.

Overlap analyses were performed for the total BCR-H repertoire and separately for IgA, IgG, and IgM repertoires. Cross-isotype sharing was also assessed by identifying identical BCR-H CDR3 sequences occurring in different immunoglobulin isotype repertoires within the same individual.

To identify candidate KD-associated shared clonotypes, BCR-H CDR3 sequences were further filtered according to their distribution across individuals. Candidate sequences were required to be absent from the healthy control group, shared by at least three KD-CAL samples and at least three KD samples, and have a clone count >10.

### Bioinformatic analysis and visualization

BCR-H CDR3 repertoire data were processed and visualized using R, the immunarch package, VDJtools, Microsoft Excel, GraphPad Prism, Adobe Illustrator, and Adobe Photoshop. Continuous variables were assessed for normality before statistical analysis. For comparisons between two independent groups, the Mann–Whitney U test was used. For comparisons among more than two independent groups, the Kruskal–Wallis test was used, followed by appropriate *post hoc* pairwise comparisons when applicable. All statistical tests were two-sided, and P < 0.05 was considered statistically significant.

## Results

### BCR repertoire characteristics, diversity, and clonal architecture in Kawasaki disease

High-throughput sequencing generated comprehensive BCR-H CDR3 repertoires from KD patients and normal controls. After MiXCR-based alignment and clonotype assembly followed by stringent filtering of productive sequences, each sample yielded a mean of 3,045,068 productive BCR-H CDR3 sequences (range, 1,479,312–4,441,988) and 353,920 clonotypes (range, 123,194–733,751). The numbers of productive sequences and clonotypes for individual samples, together with their IgA, IgG, and IgM distributions, are summarized in **Supplementary Table S3**.

The overall clonal abundance profiles were broadly comparable between KD and NC samples, with the number of clonotypes progressively decreasing as clone abundance increased (**Figure 1A**). Most samples from both groups contained approximately 200,000–400,000 clonotypes (**Figure 1B**). The inverse Simpson index integrates the richness and relative abundance of clonotypes and provides an overall measure of repertoire diversity, with lower values indicating reduced diversity and greater clonal dominance. Repertoire diversity assessed by the inverse Simpson index showed a modest reduction in KD compared with NC, while isotype-resolved analysis revealed that IgM repertoire diversity was significantly reduced in KD compared with NC (P = 0.036; **Figure 1C**), indicating preferential remodeling of the IgM-associated repertoire during acute disease.

**Figure 1:**
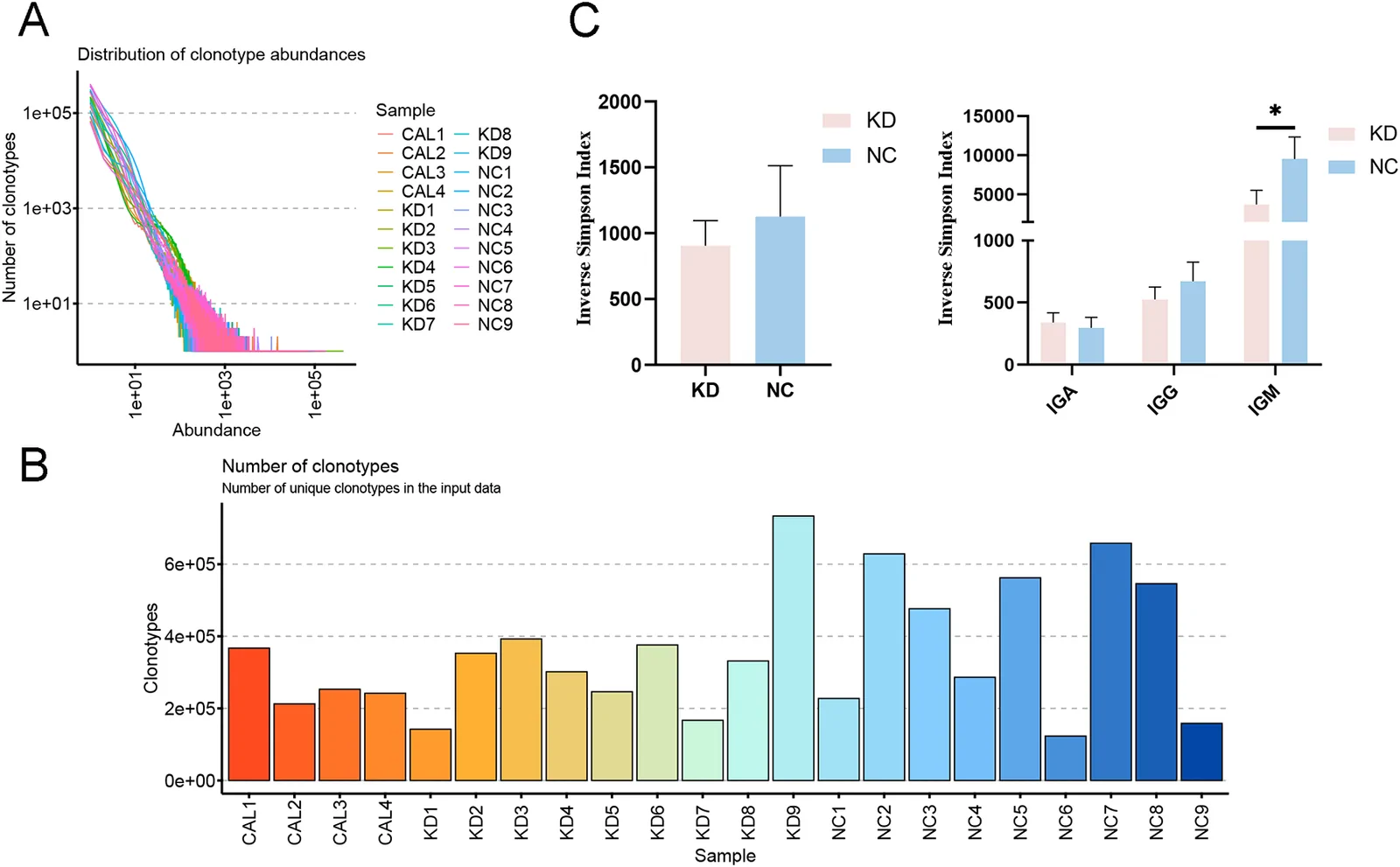
BCR-H CDR3 repertoire diversity in children with Kawasaki disease and normal controls. **(A)** Distribution of BCR-H CDR3 clonotypes according to clone abundance in individual samples (n = 22). **(B)** Number of BCR-H CDR3 clonotypes in individual samples from the KD and NC groups (n = 22). **(C)** Inverse Simpson diversity index of the overall and isotype-resolved BCR-H CDR3 repertoires in KD and NC samples (n = 22). Statistical comparisons were performed using the Mann–Whitney U test. *P < 0.05.

We next examined the clonal architecture of the BCR repertoire by ranking clonotypes according to their abundance. Compared with NCs, KD samples showed a higher proportion of the most highly ranked clonotypes, particularly within the top 1–10 and 11–100 clonotypes, whereas the proportions of clonotypes ranked 101–1,000 and 1,001–3,000 were slightly reduced (**Figure 2A**). Rare clonotypes also constituted a relatively larger proportion of the repertoire in KD than in NCs (**Figure 2B**). Consistent with these observations, analysis of repertoire space based on relative clonotype abundance demonstrated an increased contribution of high-frequency and hyperexpanded clonotypes in KD (**Figure 2C**). Notably, KD patients with CAL exhibited the most pronounced shift toward intermediate-, high-, and hyperexpanded clonotypes, with the combined contribution of these expanded populations exceeding that observed in both KD patients without CAL and NCs.

**Figure 2:**
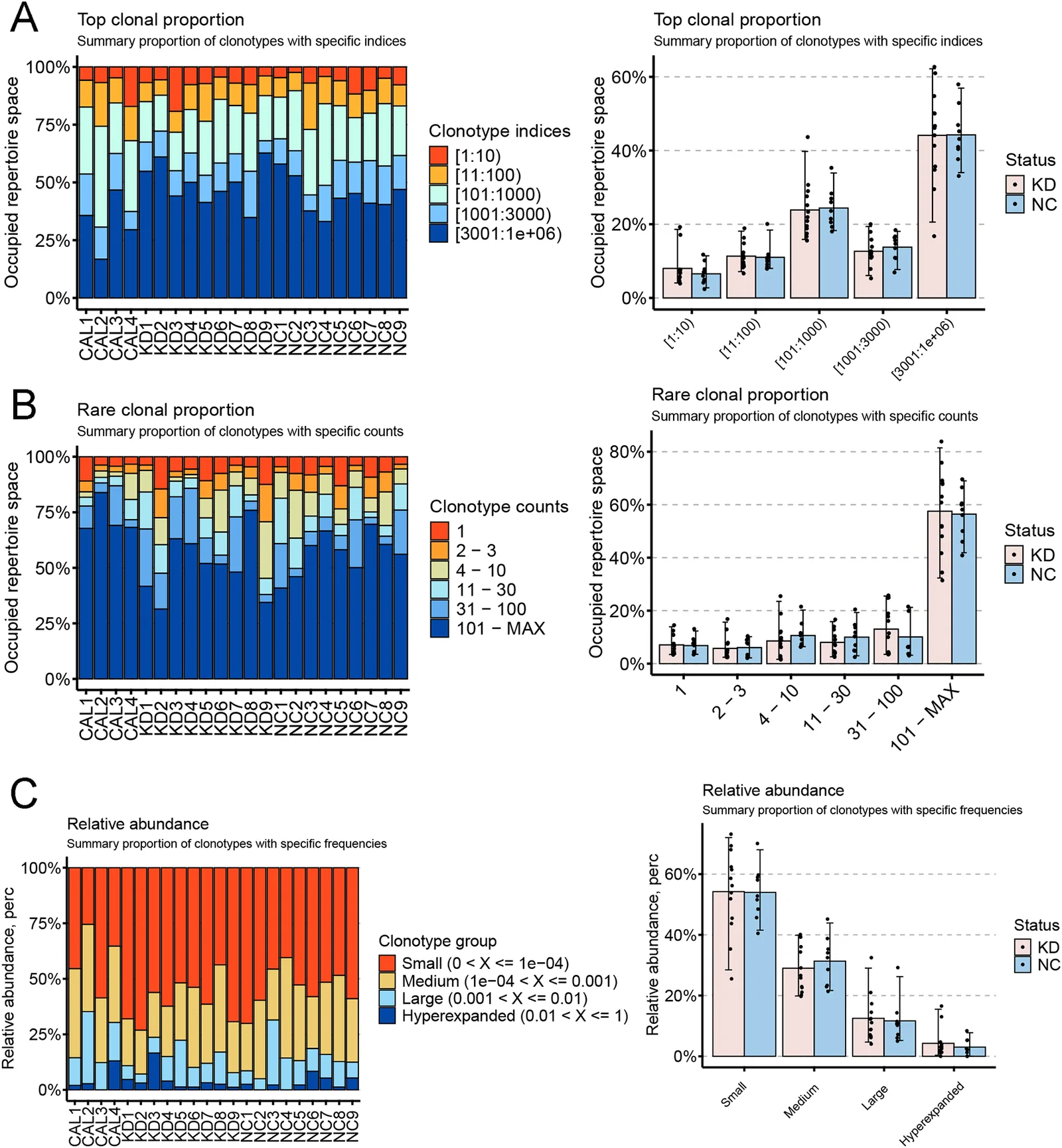
Clonal expansion and repertoire architecture of BCR-H CDR3 clonotypes in Kawasaki disease. **(A)** Distribution of BCR-H CDR3 clonotypes across rank intervals (1–10, 11–100, 101–1,000, and 1,001–3,000) in individual samples and in the KD and NC groups. **(B)** Proportion of rare clonotypes in individual samples and in the KD and NC groups. **(C)** Relative abundance of BCR-H CDR3 clonotypes across clonal expansion categories (small, medium, large, and hyperexpanded) in individual samples and in the KD and NC groups.

### Isotype-specific remodeling and immunoglobulin gene usage in Kawasaki disease

To further characterize the altered BCR repertoire, we examined clonal remodeling across immunoglobulin isotypes and immunoglobulin gene usage. The IgA repertoire showed modest increases in low-frequency and hyperexpanded clonotypes in KD compared with NCs (**Figure 3A**), while the IgG repertoire showed a greater contribution of high-frequency and hyperexpanded clonotypes in KD (**Figure 3B**). The most prominent changes were observed in the IgM repertoire, which showed a reduced proportion of low-frequency clonotypes and increased proportions of intermediate-, high-, and hyperexpanded clonotypes in KD compared with NCs (**Figure 3C**). The proportion of high-abundance IgM clonotypes was significantly increased in KD (P = 0.011; **Figure 3D**).

**Figure 3:**
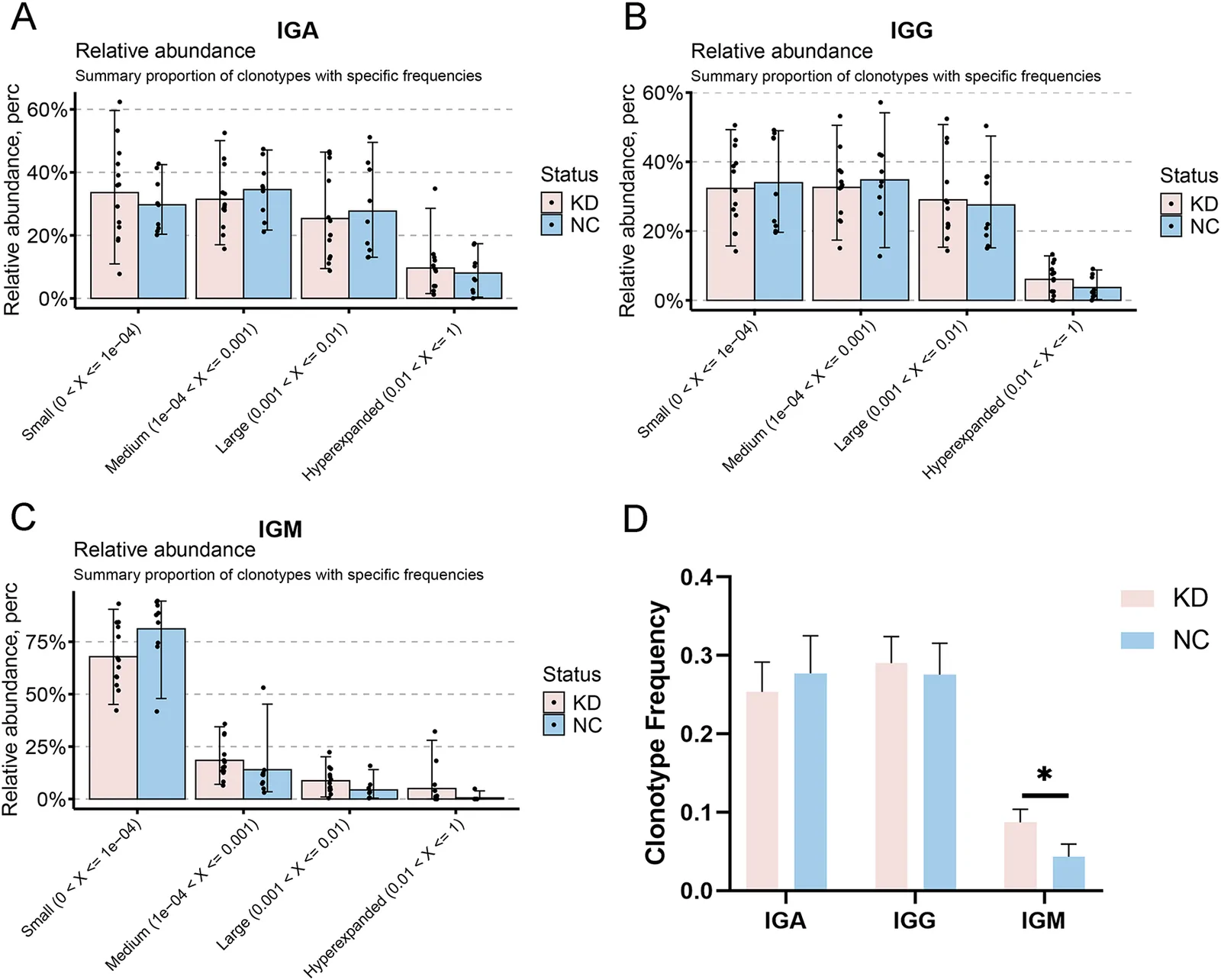
Isotype-specific clonal remodeling of the BCR-H CDR3 repertoire in Kawasaki disease. **(A)** Relative abundance distribution of IgA BCR-H CDR3 clonotypes in the KD and NC groups. **(B)** Relative abundance distribution of IgG BCR-H CDR3 clonotypes in the KD and NC groups. **(C)** Relative abundance distribution of IgM BCR-H CDR3 clonotypes in the KD and NC groups. **(D)** Comparison of the proportions of high-abundance clonotypes among IgA, IgG, and IgM BCR-H CDR3 repertoires between KD and NC groups. Statistical comparisons were performed using the Mann–Whitney U test. *P < 0.05.

Analysis of immunoglobulin gene usage further revealed selective alterations in KD. Several IGHV genes showed differential usage between groups, including increased usage of IGHV1-69, IGHV4-61, and IGHV7-4–1 and significantly reduced usage of IGHV3–11 in KD compared with NCs (P = 0.048; **Figure 4A**). IGHJ4 was the most frequently used J gene in both groups, with no significant difference between KD and NCs (**Figure 4B**). Constant-region usage showed a distinct isotype-associated pattern, with increased IGHM usage in KD, primarily among patients without CAL, whereas patients with CAL showed a relative increase in IGHA usage (**Figure 4C**).

**Figure 4:**
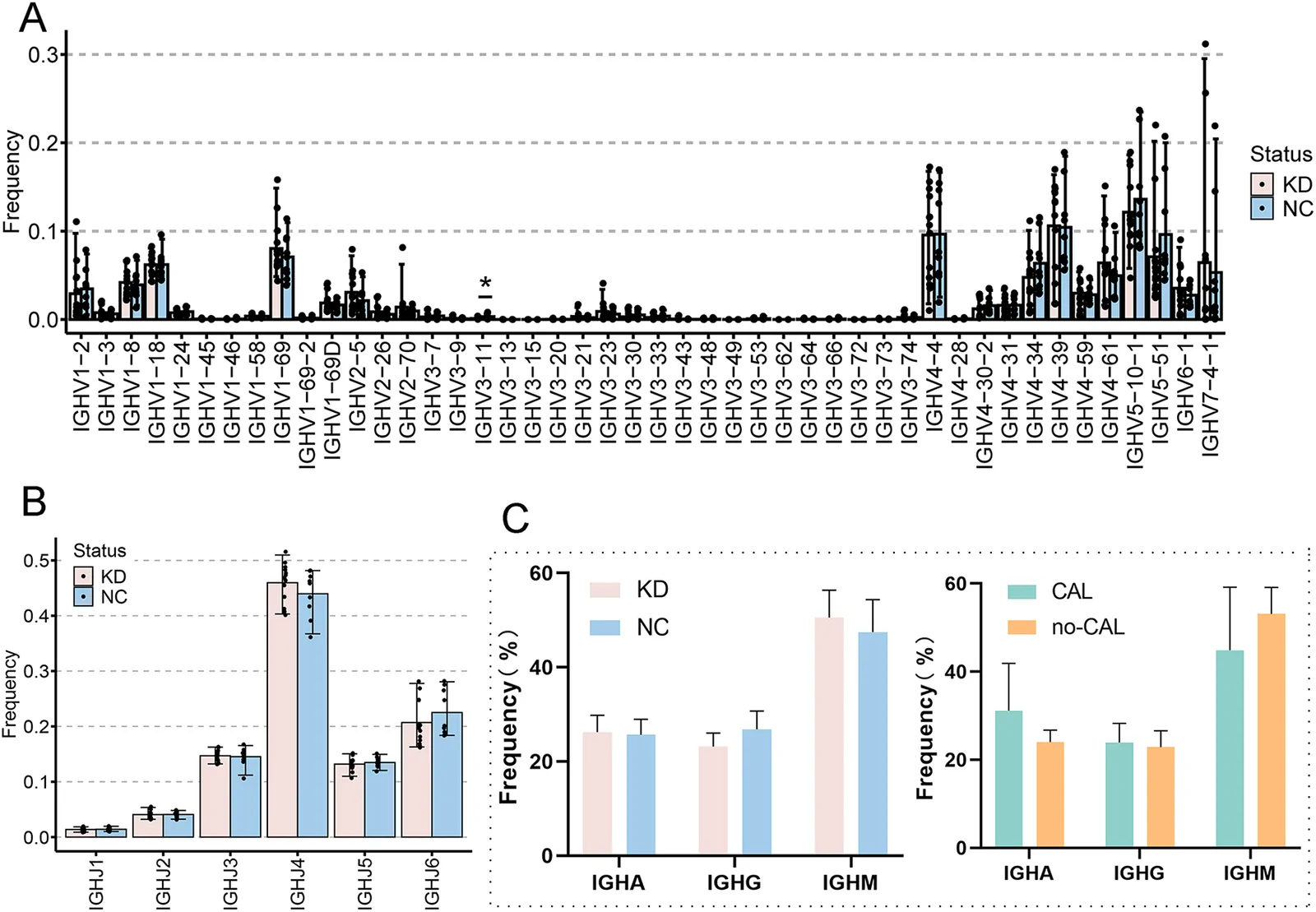
Altered immunoglobulin gene usage and CAL-associated isotype patterns in the BCR-H CDR3 repertoire of children with Kawasaki disease. **(A)** Differential usage of IGHV genes in the BCR-H CDR3 repertoires of KD and NC groups. **(B)** Differential usage of IGHJ genes in the BCR-H CDR3 repertoires of KD and NC groups. **(C)** Distribution of immunoglobulin constant-region gene usage in the KD and NC groups. *P < 0.05.

IGHV–IGHJ pairing profiles showed substantial overlap between KD and NC repertoires (**Figures 5A, B**), but several V–J combinations displayed differential usage between groups (**Figure 5C**). IGHV4-4–IGHJ4 and IGHV4-61–IGHJ4 showed increased usage in KD, whereas several combinations involving IGHV3 family genes, including IGHV3-7–IGHJ1, IGHV3-11–IGHJ2, and IGHV3-11–IGHJ5, as well as IGHV1-17–IGHJ6, were underrepresented in KD.

**Figure 5:**
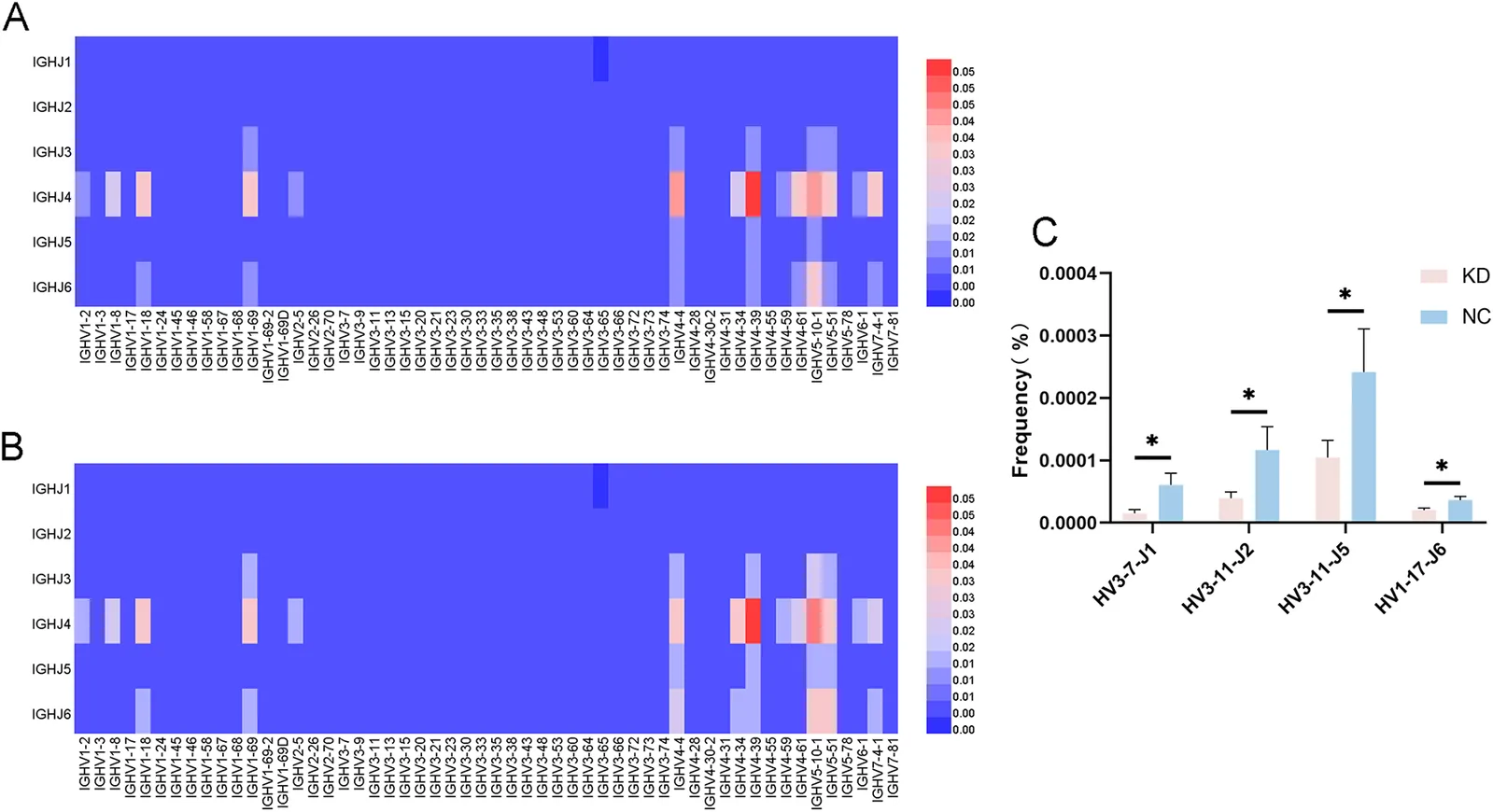
Altered IGHV–IGHJ gene-pair usage in the BCR-H CDR3 repertoire of children with Kawasaki disease. **(A)** IGHV–IGHJ pairing patterns in the BCR-H CDR3 repertoires of KD samples. **(B)** IGHV–IGHJ pairing patterns in NC samples. **(C)** Differential usage of IGHV–IGHJ gene pairs between KD and NC groups. *P < 0.05.

### CDR3 sequence characteristics in Kawasaki disease

The distribution of BCR-H CDR3 lengths was broadly similar between KD and NC groups, with both repertoires showing an approximately normal distribution centered around 14–18 amino acids (**Figure 6A**). The mean CDR3 length was also comparable between the two groups, at approximately 17.5 amino acids (**Figure 6B**). Stratification by CAL status showed a tendency toward longer CDR3 sequences in patients with CAL, with a mean length of approximately 18 amino acids, compared with less than 17.5 amino acids in patients without CAL.

**Figure 6:**
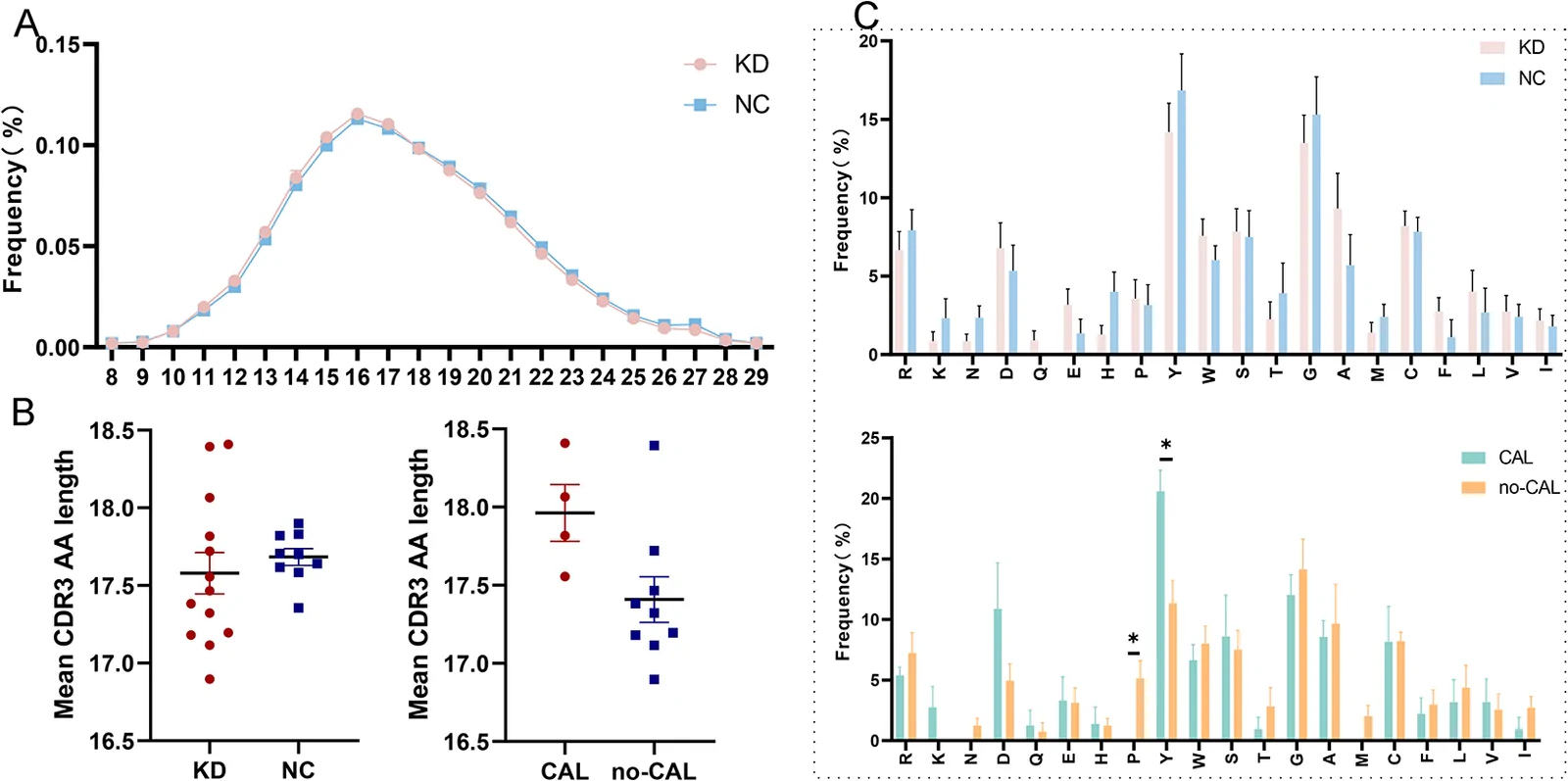
BCR-H CDR3 length and amino acid composition in Kawasaki disease, with CAL-associated differences in CDR3 amino acid usage. **(A)** Distribution of BCR-H CDR3 sequence lengths in KD and NC groups. **(B)** Mean BCR-H CDR3 sequence length in individual samples and among NC, KD without CAL, and KD with CAL groups. **(C)** Amino acid composition of BCR-H CDR3 sequences in KD and NC groups. *P < 0.05.

Glycine (G), alanine (A), tyrosine (Y), aspartic acid (D), and arginine (R) were among the most frequently used amino acids in both KD and NC repertoires (**Figure 6C**). KD samples showed modest differences in the usage of several residues, including D, tryptophan (W), and A. Notably, patients with CAL showed significantly increased usage of tyrosine (Y; P = 0.020) and decreased usage of proline (P; P = 0.034).

### Inter-individual and cross-isotype repertoire sharing

We next examined the sharing of BCR-H CDR3 sequences among individuals. Substantial repertoire overlap was observed among KD-CAL patients, with 188–4,051 shared sequences among the top 11 shared sets (**Figure 7A**). For example, 977 CDR3 sequences were shared by four CAL patients, while 3,333 were shared by three CAL patients. Similar patterns were observed across the broader KD cohort, with 896–20,628 shared sequences among the top 30 shared sets and 2,077 CDR3 sequences shared by seven KD patients (**Figure 7B**).

**Figure 7:**
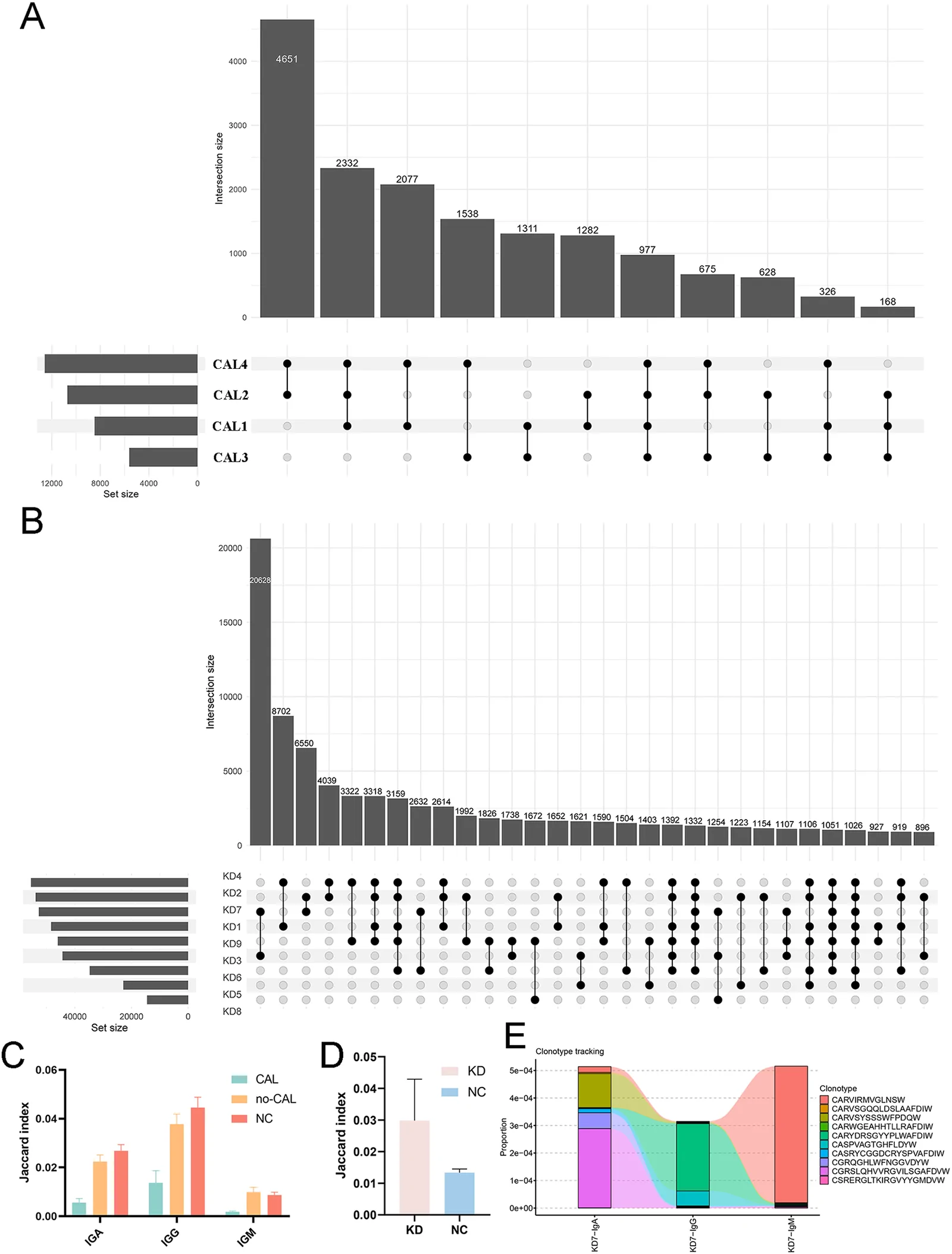
Inter-individual and cross-isotype sharing of BCR-H CDR3 clonotypes in Kawasaki disease. **(A)** Number of shared BCR-H CDR3 sequences among KD patients with CAL based on the top 30 shared clonotypes. **(B)** Number of shared BCR-H CDR3 sequences among KD patients without CAL based on the top 30 shared clonotypes. **(C)** Comparison of Jaccard similarity indices for BCR-H CDR3 repertoires across different immunoglobulin isotypes among NC, KD without CAL, and KD with CAL groups. **(D)** Comparison of cross-isotype BCR-H CDR3 overlap indices among NC, KD without CAL, and KD with CAL groups. **(E)** Representative example of shared BCR-H CDR3 sequences across IgA, IgG, and IgM repertoires in an individual KD patient (KD7).

Isotype-resolved analysis showed that IgG had the highest inter-individual overlap, followed by IgA and IgM (**Figure 7C**). Overall, repertoire overlap tended to be greater in KD than in NCs. We further identified 20 candidate KD-associated CDR3 sequences that were absent from NCs but shared by at least three KD-CAL patients and three KD patients overall, with a clone count >10 (**Table 1**).

**Table 1:** Candidate Kawasaki disease-associated BCR-H CDR3 clonotypes shared among patients with coronary artery lesions and other KD patients but absent from healthy controls.

IGHV	IGHD	IGHJ	CDR3 sequence	KD(%)	NC(%)
IGHV1-2	IGHD1-7	IGHJ2	CARKPVGNWYFDLW	5.049	0
IGHV4-61	IGHD3-3	IGHJ4	CARELSGWAPFDYW	11.937	0
IGHV5-51	IGHD3-10	IGHJ4	CARRGNLYGSGSYGNFDYW	2.138	0
IGHV7-4-1	IGHD1-1	IGHJ4	CVRGGHNCDYW	0.784	0
IGHV5-51	IGHD3-10	IGHJ4	CARVMVRGDSSGWSYYFDYW	0.557	0
IGHV5-51	IGHD1-7	IGHJ3	CARRNLGMAPGAFDIW	0.611	0
IGHV5-51	IGHD4-17	IGHJ4	CASSLPTYGDFYFDYW	0.413	0
IGHV5-51	IGHD2-15	IGHJ5	CARRGGPVVFWFDPW	0.058	0
IGHV5-10-1	IGHD6-13	IGHJ6	CARHSDSRGYVAAAGFYYYYHMDVW	0.009	0
IGHV1-69	IGHD5-12	IGHJ4	CARERYTGYGPWDHW	0.731	0
IGHV4-39	IGHD2-21	IGHJ3	CARHWAVADAFDIW	0.203	0
IGHV6-1	IGHD6-19	IGHJ5	CARDQAVAGTLINWFDPW	4.569	0
IGHV5-51	IGHD3-10	IGHJ4	CARLQRGSGRVIEYW	1.564	0
IGHV4-61	IGHD1-26	IGHJ4	CARSGGQRTFDYW	0.370	0
IGHV4-4	IGHD5-24	IGHJ6	CARRGYNFNYYYYYMDVW	0.006	0
IGHV5-51	IGHD3-10	IGHJ4	CARRGPGNNVPGNYW	0.039	0
IGHV4-61	IGHD3-22	IGHJ4	CARVAQFDTSGYTFDTW	0.406	0
IGHV4-34	IGHD1-26	IGHJ4	CARGQYYFDYW	0.001	0
IGHV5-10-1	IGHD3-10	IGHJ3	CARLTGDAFDIW	0.004	0
IGHV4-59	IGHD2-2	IGHJ3	CARVGADAFDIW	0.001	0

We also assessed sharing of identical BCR-H CDR3 sequences across immunoglobulin isotypes within individuals. Cross-isotype overlap was higher in KD-CAL than in NCs (**Figure 7D**), and several CDR3 sequences were shared across IgA, IgG, and IgM in the representative KD7 sample (**Figure 7E**). Together, these findings demonstrate extensive inter-individual and cross-isotype BCR sharing in KD, with greater convergence observed in patients with coronary artery involvement.

## Discussion

Our findings demonstrate that acute KD is associated with substantial remodeling of the peripheral BCR repertoire. Although the overall clonotype abundance distribution remained broadly comparable between KD and healthy controls, KD patients showed reduced repertoire diversity together with enrichment of highly ranked and hyperexpanded clonotypes, indicating a shift toward a less even repertoire dominated by selected B-cell clones. This pattern is consistent with previous observations that antigen exposure or infection can reshape BCR repertoires through selective clonal expansion rather than uniform activation of the entire B-cell compartment (18). In KD, Ko et al. reported reduced IgM repertoire diversity and prominent expansion of specific IgM clonotypes during the acute phase, further supporting disease-associated B-cell selection (19). More broadly, convergent and expanded BCR populations have been recognized as characteristic features of antigen-driven immune responses (20). Thus, our findings suggest that acute KD induces a selective restructuring of the B-cell repertoire, potentially reflecting the recruitment and expansion of B-cell clones responding to disease-associated antigenic or inflammatory stimuli.

The IgM compartment showed the most pronounced repertoire remodeling in acute KD. Compared with healthy controls, KD patients exhibited reduced IgM diversity, depletion of low-frequency clonotypes, and enrichment of intermediate-, high-frequency, and hyperexpanded clonotypes, together with a significant increase in highly abundant IgM clones. Early studies demonstrated increased IgM-bearing peripheral B cells and IgM-secreting cells during acute KD (21, 22), while subsequent work showed enhanced IgM production associated with increased B-cell growth factors and IL-6 activity (23). Changes in circulating immunoglobulin-bearing B-cell subsets during acute KD further indicated dynamic remodeling of peripheral B-cell compartments (24). At the clonal level, IgM-producing B cells showed oligoclonal VH expansion and recurrent sequences, supporting selective B-cell activation (25). Acute KD B cells also exhibited increased IgM production and inflammatory activation following TLR9 stimulation (26), consistent with heightened responsiveness of the IgM compartment. Most importantly, high-throughput BCR sequencing demonstrated reduced IgM diversity and expansion of dominant IgM clonotypes in acute KD, with many expanded clones disappearing after IVIG treatment (19). Single-cell profiling has further confirmed extensive B-cell and antibody-secreting cell remodeling during acute KD (27). Together, these findings indicate that acute KD-associated IgM responses involve selective repertoire remodeling rather than simply increased IgM production, with preferential expansion of a restricted set of dominant B-cell clones.

The relative enrichment of IGHA usage in CAL patients suggests that coronary artery involvement may be associated with selective remodeling of the IgA-associated B-cell repertoire. Previous pathological studies have consistently demonstrated prominent IgA plasma-cell responses in KD, including infiltration of the coronary arterial wall and other inflamed tissues (10, 28). Importantly, V(D)J analysis of coronary tissue demonstrated an oligoclonal IgA response, supporting antigen-driven selection rather than nonspecific B-cell activation (29). Clinical evidence further links IgA responses to coronary involvement: in a cohort of 241 KD patients, acute-phase IgA levels were significantly higher in patients with CALs and increased with coronary aneurysm size (12). Together, these findings and our observation of enhanced IgA-associated clonal remodeling suggest that CAL may be accompanied by a distinct humoral response characterized by selective B-cell expansion and IgA enrichment, although whether these clones directly contribute to coronary vascular injury remains to be established.

The extensive sharing of BCR-H CDR3 sequences among KD patients, together with the identification of recurrent clonotypes absent from healthy controls, suggests that the humoral response in KD is not entirely individualized but may contain a convergent component shaped by shared antigenic stimuli. This interpretation is supported by previous repertoire studies showing recurrent IgM CDR3 clonotypes among multiple patients with acute KD and oligoclonal expansion of IgM-producing B cells with highly similar VH sequences across patients (19, 25). More direct evidence has come from plasmablast-derived monoclonal antibodies, in which antibodies from multiple KD patients recognized a common protein epitope and shared a characteristic CDR3 motif, demonstrating a convergent antibody response (30). Recent work further identified a common antigenic target recognized by monoclonal antibodies from children with acute KD across fatal cases spanning several decades (31). Together, these findings support the possibility that the recurrent CDR3 sequences identified in our cohort represent clonotypes preferentially selected by shared KD-associated antigenic stimulation. However, their disease specificity and antigen recognition remain to be established experimentally.

The increased sharing of identical BCR-H CDR3 sequences across IgM, IgG, and IgA repertoires in KD, particularly in patients with CAL, may indicate clonal relationships among different immunoglobulin compartments. Consistent with this interpretation, previous analysis of acute KD repertoires showed that dominant IgM CDR3 clonotypes were frequently detected in IgG and IgA repertoires, indicating that selected B-cell clones can be represented across multiple isotypes during the disease response (19). Studies of human BCR repertoires have similarly demonstrated clonally related sequences carrying identical or highly similar V(D)J and CDR3 regions across different immunoglobulin subclasses, consistent with isotype switching and diversification of antigen-experienced B-cell clones (32, 33). In KD, the detection of clonally expanded plasmablast-derived antibodies further supports active B-cell differentiation during the acute response (30, 34). The greater cross-isotype sharing observed in CAL patients may therefore reflect more extensive expansion and diversification of selected B-cell clones associated with coronary involvement. Nevertheless, because our bulk BCR sequencing does not directly establish cellular lineage or the temporal order of class switching, these findings should be interpreted as evidence consistent with shared clonal origins rather than direct evidence of individual class-switch trajectories.

Several limitations should be acknowledged. First, the relatively small cohort, particularly the CAL subgroup, limits statistical power and warrants validation in larger, independent cohorts. Second, although recurrent and cross-isotype CDR3 sequences suggest convergent and clonally related B-cell responses, their antigen specificity and functional relevance to coronary artery injury were not experimentally validated. Third, bulk BCR sequencing cannot resolve cellular lineage or directly determine the temporal trajectory of class-switch recombination. Importantly, although the identified repertoire features may provide potential biomarkers for KD-associated immune responses, their diagnostic value has not been established, and their ability to distinguish KD from other febrile or inflammatory conditions remains unknown. Future studies integrating larger disease-control cohorts, paired single-cell BCR sequencing, antigen-binding assays, and longitudinal sampling will be important to evaluate the diagnostic utility and biological significance of these KD-associated B-cell clones.

Overall, our study shows that acute KD is characterized by coordinated remodeling of the peripheral BCR repertoire, including reduced diversity, expansion of dominant clonotypes, recurrent CDR3 sequences, and increased cross-isotype sharing. These features were particularly evident in the IgM compartment and in patients with CAL, while the enrichment of IgA-associated responses and recurrent clonotypes suggests that selected B-cell populations may contribute to the humoral response associated with coronary involvement. Although the antigen specificity and functional significance of these clonotypes remain to be determined, our findings provide a repertoire-level framework for investigating convergent and clonally related B-cell responses in KD.

## Data Availability

The datasets presented in this study can be found in online repositories (https://zenodo.org/records/23226879).

## References

[1] Jone PN Tremoulet A Choueiter N Dominguez SR Harahsheh AS Mitani Y . Update on diagnosis and management of Kawasaki disease: A scientific statement from the American Heart Association. Circulation. (2024) 150:e481–500. doi: 10.1161/CIR.0000000000001295 39534969

[2] Goel AR Yalcindag A . An update on Kawasaki disease. Curr Rheumatol Rep. (2024) 27:4. doi: 10.1007/s11926-024-01167-4 39625646

[3] Hara T Sakai Y . The etiopathogenesis of Kawasaki disease: Evolving understanding of diverse triggers. Immun Inflammation Dis. (2025) 13:e70267. doi: 10.1002/iid3.70267 41017238 PMC12477333

[4] Agrafiotou A Sapountzi E Margoni A Fotis L . Immunophenotype of Kawasaki disease: Insights into pathogenesis and treatment response. Life (Basel). (2025) 15. doi: 10.3390/life15071012 40724515 PMC12299537

[5] Okada S Sakai A Ohnishi Y Yasudo H Motonaga T Fukano R . Necrotic change of tunica media plays a key role in the development of coronary artery lesions in Kawasaki disease. Circ J. (2024) 88:1709–14. doi: 10.1253/circj.CJ-24-0295 39111854

[6] Kim TH Son Y Lee H Lee K Lee H Park J . Biomarkers and related factors for the diagnosis, risk of coronary artery lesions, and resistance to intravenous immunoglobulin in Kawasaki disease: An umbrella review of meta-analyses. Pediatr Cardiol. (2025) 46:1488–504. doi: 10.1007/s00246-024-03563-0 38980324

[7] Akay N Torunoglu Z Aslan E Gul U Kilic Konte E Kalay Senturk NG . Predictors of severe coronary involvement in children with Kawasaki disease: A single-centre cohort study. Cardiol Young. (2026) 36(5):1035–44. doi: 10.1017/S1047951126113304 42219926

[8] Nait-Ladjemil D Harahsheh AS Choueiter N Agha HM Alrabte H Al-Saoud SYA . Pharmacoeconomic analysis and considerations for the management of Kawasaki disease in the Arab countries-a multinational, multi-institutional project of the Kawasaki Disease Arab Initiative (Kawarabi) (a project methodology paper). Turk Arch Pediatr. (2025) 60:172–81. doi: 10.5152/TurkArchPediatr.2025.24248 40094277 PMC11963343

[9] Lin Q Lv X Qiu Q Mei L Chen L Song S . Delineation of single-cell atlas provides new insights for development of coronary artery lesions in Kawasaki disease: Bad and good signaling molecules. Front Pediatr. (2025) 13:1596643. doi: 10.3389/fped.2025.1596643 40636515 PMC12237900

[10] Rowley AH Eckerley CA Jack HM Shulman ST Baker SC . IgA plasma cells in vascular tissue of patients with Kawasaki syndrome. J Immunol. (1997) 159:5946–55. doi: 10.4049/jimmunol.159.12.5946 9550392

[11] Rowley AH Shulman ST Garcia FL Guzman-Cottrill JA Miura M Lee HL . Cloning the arterial IgA antibody response during acute Kawasaki disease. J Immunol. (2005) 175:8386–91. doi: 10.4049/jimmunol.175.12.8386 16339580

[12] Kim JJ Kim HJ Yu JJ Yun SW Lee KY Yoon KL . IgA levels are associated with coronary artery lesions in Kawasaki disease. Korean Circ J. (2021) 51:267–78. doi: 10.4070/kcj.2020.0345 33655727 PMC7925970

[13] Inoue T Baba Y Kurosaki T . BCR signaling in germinal center B cell selection. Trends Immunol. (2024) 45:693–704. doi: 10.1016/j.it.2024.07.005 39168721

[14] Kuo HC Pan CT Huang YH Huang FC Lin YS Li SC . Global investigation of immune repertoire suggests Kawasaki disease has infectious cause. Circ J. (2019) 83:2070–8. doi: 10.1253/circj.CJ-19-0206 31378745

[15] Ma J Song X Li X Tang Y Lv H Chen Y . Comprehensive immune repertoire profiling reveals distinct characteristics in Kawasaki disease and a mouse model. J Immunol. (2026) 215(2):vkaf369. doi: 10.1093/jimmun/vkaf369 41764728

[16] Wang Q Feng D Jia S Lu Q Zhao M . B-cell receptor repertoire: Recent advances in autoimmune diseases. Clin Rev Allergy Immunol. (2024) 66:76–98. doi: 10.1007/s12016-024-08984-6 38459209

[17] Raybould MIJ Rees AR Deane CM . Current strategies for detecting functional convergence across B-cell receptor repertoires. MAbs. (2021) 13:1996732. doi: 10.1080/19420862.2021.1996732 34781829 PMC8604390

[18] Hoehn KB Fowler A Lunter G Pybus OG . The diversity and molecular evolution of B-cell receptors during infection. Mol Biol Evol. (2016) 33:1147–57. doi: 10.1093/molbev/msw015 26802217 PMC4839220

[19] Ko TM Kiyotani K Chang JS Park JH Yin Yew P Chen YT . Immunoglobulin profiling identifies unique signatures in patients with Kawasaki disease during intravenous immunoglobulin treatment. Hum Mol Genet. (2018) 27:2671–7. doi: 10.1093/hmg/ddy176 29771320 PMC6048982

[20] Miho E Roskar R Greiff V Reddy ST . Large-scale network analysis reveals the sequence space architecture of antibody repertoires. Nat Commun. (2019) 10:1321. doi: 10.1038/s41467-019-09278-8 30899025 PMC6428871

[21] Kuno-Sakai H Ohta T Kikuchi T Hikino N Takesue R Iwagaki H . Polyclonal increases of serum immunoglobulins and increase of immunoglobulin-bearing peripheral blood lymphocytes in children with Kawasaki disease. Tokai J Exp Clin Med. (1982) 7:661–4. 7184197

[22] Kawamori J Miyake T Yoshida T . B-cell function in Kawasaki disease and the effect of high-dose gamma-globulin therapy. Acta Paediatr Jpn. (1989) 31:537–43. doi: 10.1111/j.1442-200x.1989.tb01351.x 2515730

[23] Ding XT Yang XQ Li CR Shen J . Immunologic abnormalities in children with acute Kawasaki disease. Chin Med J (Engl). (1993) 106:688–92. 8287706

[24] Shingadia D O'Gorman M Rowley AH Shulman ST . Surface and cytoplasmic immunoglobulin expression in circulating B-lymphocytes in acute Kawasaki disease. Pediatr Res. (2001) 50:538–43. doi: 10.1203/00006450-200110000-00019 11568300

[25] Lee HH Park IH Shin JS Kim DS . Immunoglobulin V(H) chain gene analysis of peripheral blood IgM-producing B cells in patients with Kawasaki disease. Yonsei Med J. (2009) 50:493–504. doi: 10.3349/ymj.2009.50.4.493 19718396 PMC2730610

[26] Giordani L Quaranta MG Marchesi A Straface E Pietraforte D Villani A . Increased frequency of immunoglobulin (Ig)A-secreting cells following toll-like receptor (TLR)-9 engagement in patients with Kawasaki disease. Clin Exp Immunol. (2011) 163:346–53. doi: 10.1111/j.1365-2249.2010.04297.x 21175593 PMC3048618

[27] Wang Z Xie L Ding G Song S Chen L Li G . Single-cell RNA sequencing of peripheral blood mononuclear cells from acute Kawasaki disease patients. Nat Commun. (2021) 12:5444. doi: 10.1038/s41467-021-25771-5 34521850 PMC8440575

[28] Rowley AH Shulman ST Mask CA Finn LS Terai M Baker SC . IgA plasma cell infiltration of proximal respiratory tract, pancreas, kidney, and coronary artery in acute Kawasaki disease. J Infect Dis. (2000) 182:1183–91. doi: 10.1086/315832 10979916

[29] Rowley AH Shulman ST Spike BT Mask CA Baker SC . Oligoclonal IgA response in the vascular wall in acute Kawasaki disease. J Immunol. (2001) 166:1334–43. doi: 10.4049/jimmunol.166.2.1334 11145718

[30] Rowley AH Arrollo D Shulman ST Torres A O'Brien A Wylie K . Analysis of plasmablasts from children with Kawasaki disease reveals evidence of a convergent antibody response to a specific protein epitope. J Infect Dis. (2023) 228:412–21. doi: 10.1093/infdis/jiad048 36808252 PMC10428203

[31] Rowley AH Byrd R Arrollo D O'Brien A Shulman S Terai M . Monoclonal antibodies from children with acute Kawasaki disease identify a common antigenic target in fatal cases over 5 decades. Lab Invest. (2025) 105:104131. doi: 10.1016/j.labinv.2025.104131 40122524 PMC12822914

[32] H IJ van Schouwenburg PA van Zessen D Pico-Knijnenburg I Driessen GJ Stubbs AP . Evaluation of the antigen-experienced B-cell receptor repertoire in healthy children and adults. Front Immunol. (2016) 7:410. doi: 10.3389/fimmu.2016.00410 27799928 PMC5066086

[33] Kitaura K Yamashita H Ayabe H Shini T Matsutani T Suzuki R . Different somatic hypermutation levels among antibody subclasses disclosed by a new next-generation sequencing-based antibody repertoire analysis. Front Immunol. (2017) 8:389. doi: 10.3389/fimmu.2017.00389 28515723 PMC5413556

[34] Rowley AH Baker SC Arrollo D Gruen LJ Bodnar T Innocentini N . A protein epitope targeted by the antibody response to Kawasaki disease. J Infect Dis. (2020) 222:158–68. doi: 10.1093/infdis/jiaa066 32052021 PMC7296860

