# Supplementary material for: B-cell receptor repertoire remodeling in Kawasaki disease is associated with clonal expansion and distinct repertoire features in coronary artery lesions
Source: Front Immunol. 2026 Oct 14;17:1994376. doi: 10.3389/fimmu.2026.1994376 (PMC13713354; doi:10.3389/fimmu.2026.1994376)
Supplement: Supplementary file 1 [file Table1.docx]

**Supplementary Table S1. Clinical and laboratory characteristics of patients with Kawasaki disease**

| **Sample** | KD-CAL1 | KD-CAL2 | KD-CAL3 | KD-CAL4 | KD1 | KD2 | KD3 | KD4 | KD5 | KD6 | KD7 | KD8 | KD9 |
| --- | --- | --- | --- | --- | --- | --- | --- | --- | --- | --- | --- | --- | --- |
| **Sex** | Female | Male | Female | Male | Male | Male | Male | Male | Male | Female | Male | Female | Male |
| **Age(years)** | 6.58 | 2.25 | 1.75 | 10.05 | 5.17 | 3.25 | 6.50 | 3.25 | 0.49 | 2.00 | 1.50 | 4.17 | 3.00 |
| **KD type** | cKD | cKD | iKD | cKD | cKD | cKD | cKD | cKD | cKD | cKD | cKD | cKD | iKD |
| **WBC (×10⁹/L)** | 20.16 | 28.3 | 13.99 | 19.07 | 12.28 | 33.15 | 10.77 | 15.04 | 22.95 | 12.03 | 8.49 | 26.11 | 26.66 |
| **Platelet count (×10⁹/L)** | 239 | 473 | 639 | 231 | 248 | 170 | 192 | 286 | 310 | 333 | 490 | 449 | 429 |
| **Absolute lymphocyte count (×10⁹/L)** | 1.24 | 2.81 | 5.19 | 0.8 | 5.38 | 3.07 | 2.17 | 5.84 | 5.3 | 4.2 | 4.74 | 4.7 | 1.97 |
| **Absolute neutrophil count (×10⁹/L)** | 18.07 | 24.09 | 7.34 | 16.84 | 5.33 | 32.65 | 8.02 | 8.13 | 17.21 | 6.18 | 2.37 | 19.99 | 23.51 |
| **Absolute monocyte count (×10⁹/L)** | 0.8 | 1.02 | 1.03 | 1.2 | 0.95 | 2.04 | 0.38 | 0.68 | 0.04 | 1.33 | 0.84 | 1.17 | 1.15 |
| **Absolute eosinophil count (×10⁹/L)** | 0.05 | 0.16 | 0.3 | 0.1 | 0.51 | 0.01 | 0.11 | 0.3 | 0.34 | 0.3 | 0.52 | 0.13 | 0.02 |
| **Absolute basophil count (×10⁹/L)** | 0 | 0 | 0.03 | 0.01 | 0.02 | 0.01 | 0.02 | 0.02 | 0.01 | 0.02 | 0.02 | 0.02 | 0.01 |
| **Lymphocyte (%)** | 6.2 | 9.9 | 37.1 | 4.2 | 43.8 | 8 | 20.1 | 38.8 | 23.1 | 34.9 | 55.8 | 18 | 7.4 |
| **Neutrophil (%)** | 89.6 | 85.2 | 52.5 | 88.3 | 43.4 | 85.7 | 74.6 | 54.1 | 75 | 51.3 | 28 | 76.5 | 88.2 |
| **IgA** | 1.57 | 0.54 | 0.47 | 1.21 | 0.06 | 0.83 | 0.48 | 0.67 | 0.19 | 0.24 | 1.56 | 1.16 | 1.05 |
| **IgM** | 1.65 | 0.99 | 1.9 | 1.23 | 1.02 | 2.03 | 0.95 | 1.02 | 0.4 | 1.17 | 1.96 | 0.8 | 1.28 |
| **IgG** | 5.08 | 4.52 | 4.5 | 6.27 | 7.42 | 6.68 | 5.43 | 31.01 | 3.46 | 4.11 | 6.33 | 9.14 | 7.9 |
| **Left coronary artery (mm)** | 3.4 | 3.8 | 2.8 | 3.8 | 2.2 | 2.4 | 2.6 | 2.3 | 2 | 2.2 | 2.1 | 2.8 | 2.3 |
| **Right coronary artery (mm)** | 3.5 | 2.6 | 2.7 | 2.9 | 2.1 | 2.2 | 2.3 | 1.9 | 2.2 | 1.4 | 2 | 2.8 | 2 |
| **days post fever onset(d)** | 6 | 5 | 7 | 6 | 3 | 2 | 3 | 3 | 5 | 4 | 3 | 6 | 2 |

**KD, Kawasaki disease; CAL, coronary artery lesion; WBC, white blood cell count; CRP, C-reactive protein; IgA, immunoglobulin A; IgM, immunoglobulin M; IgG, immunoglobulin G; cKD, Complete KD; iKD, incomplete KD.**

**Supplementary Table S2** Human BCR-H CDR3 primer sequence (5'-3')

| Primer name | Primer type | Primer（5'-3'） |
| --- | --- | --- |
| HV1 | Forward | GCACAGCCTACATGGAGCTGAG |
| HV2 | Forward | CACCATCTCCAAGGACACCTCC |
| HV3 | Forward | GXTTCACCATCTCCAGAGA |
| HV4 | Forward | CCTGAAGCTGAGCTCTGTGAC |
| HV5 | Forward | TGCAGTGGAGCAGCCTGAAGG |
| HV6 | Forward | CCCTGCAGCTGAACTCTGTGAC |
| HV7 | Forward | GTATGCCCAGGGCTTCACAGG |
| IGHC-A | Reverse | GAAGACCTTGGGGCTGGTCG |
| IGHC-G | Reverse | GAAGACCGATGGGCCCTTGGTG |
| IGHC-M | Reverse | CAGGAGACGAGGGGGAAAAGG |

**Supplementary Table S3 BCR-H CDR3 receptor library HTS Productive and Clonotype number and subtype classification data table**

| Group | sample | IGH | | Subclass | | | |
| --- | --- | --- | --- | --- | --- | --- | --- |
|  |  | Productive | Clonotype | Name | Productive | Clonotype | Clonotype/  Productive |
| NC | NC1 | 2510077 | 227556 | IGA | 754749 | 48513 | 6.43% |
|  |  |  |  | IGG | 1092491 | 75031 | 6.87% |
|  |  |  |  | IGM | 662837 | 104011 | 15.69% |
| NC | NC2 | 4105773 | 628867 | IGA | 1237202 | 80984 | 6.55% |
|  |  |  |  | IGG | 986215 | 79756 | 8.09% |
|  |  |  |  | IGM | 1882355 | 467925 | 24.86% |
| NC | NC3 | 3398093 | 476484 | IGA | 2088705 | 174889 | 8.37% |
|  |  |  |  | IGG | 920409 | 144440 | 15.69% |
|  |  |  |  | IGM | 388979 | 157154 | 40.40% |
| NC | NC4 | 3532993 | 286391 | IGA | 1331035 | 71986 | 5.41% |
|  |  |  |  | IGG | 1306918 | 65769 | 5.03% |
|  |  |  |  | IGM | 895040 | 148635 | 16.61% |
| NC | NC5 | 2886420 | 562405 | IGA | 1210733 | 135417 | 11.18% |
|  |  |  |  | IGG | 1137889 | 127123 | 11.17% |
|  |  |  |  | IGM | 537798 | 299864 | 55.76% |
| NC | NC6 | 1640977 | 123194 | IGA | 849948 | 51960 | 6.11% |
|  |  |  |  | IGG | 742463 | 57445 | 7.74% |
|  |  |  |  | IGM | 48566 | 13788 | 28.39% |
| NC | NC7 | 4441988 | 658123 | IGA | 1673013 | 116312 | 6.95% |
|  |  |  |  | IGG | 1691022 | 121117 | 7.16% |
|  |  |  |  | IGM | 1077953 | 420693 | 39.03% |
| NC | NC8 | 3950554 | 546023 | IGA | 1656605 | 97409 | 5.88% |
|  |  |  |  | IGG | 1102185 | 79797 | 7.24% |
|  |  |  |  | IGM | 1191764 | 368816 | 30.95% |
| NC | NC9 | 2413448 | 158529 | IGA | 1148996 | 53068 | 4.62% |
|  |  |  |  | IGG | 1074288 | 64077 | 5.96% |
|  |  |  |  | IGM | 190164 | 41383 | 21.76% |
| KD | KD1 | 1809470 | 142231 | IGA | 607529 | 40341 | 6.64% |
|  |  |  |  | IGG | 711392 | 49676 | 6.98% |
|  |  |  |  | IGM | 490549 | 52213 | 10.64% |
| KD | KD2 | 1504636 | 352884 | IGA | 567506 | 88570 | 15.61% |
|  |  |  |  | IGG | 538604 | 83771 | 15.55% |
|  |  |  |  | IGM | 398526 | 180542 | 45.30% |
| KD | KD3 | 4415178 | 392661 | IGA | 1874154 | 106058 | 5.66% |
|  |  |  |  | IGG | 948215 | 64032 | 6.75% |
|  |  |  |  | IGM | 1592809 | 222570 | 13.97% |
| KD | KD4 | 3874599 | 301665 | IGA | 1061664 | 66030 | 6.22% |
|  |  |  |  | IGG | 1972509 | 95681 | 4.85% |
|  |  |  |  | IGM | 840426 | 139953 | 16.65% |
| KD | KD5 | 1479312 | 246173 | IGA | 587278 | 86318 | 14.70% |
|  |  |  |  | IGG | 784364 | 102491 | 13.07% |
|  |  |  |  | IGM | 107670 | 57363 | 53.28% |
| KD | KD6 | 2561127 | 375919 | IGA | 813635 | 54243 | 6.67% |
|  |  |  |  | IGG | 604488 | 49813 | 8.24% |
|  |  |  |  | IGM | 1143004 | 271862 | 23.78% |
| KD | KD7 | 2223586 | 166928 | IGA | 955405 | 55235 | 5.78% |
|  |  |  |  | IGG | 498372 | 36434 | 7.31% |
|  |  |  |  | IGM | 769809 | 75258 | 9.78% |
| KD | KD8 | 3908890 | 331532 | IGA | 1730242 | 68237 | 3.94% |
|  |  |  |  | IGG | 1063703 | 46853 | 4.40% |
|  |  |  |  | IGM | 1114945 | 216441 | 19.41% |
| KD | KD9 | 2966502 | 733751 | IGA | 816790 | 78680 | 9.63% |
|  |  |  |  | IGG | 512321 | 60132 | 11.74% |
|  |  |  |  | IGM | 1637388 | 594899 | 36.33% |
| KD | CAL1 | 2604495 | 367240 | IGA | 1593366 | 173821 | 10.91% |
|  |  |  |  | IGG | 887584 | 114401 | 12.89% |
|  |  |  |  | IGM | 123545 | 79017 | 63.96% |
| KD | CAL2 | 3703686 | 212645 | IGA | 2662071 | 110306 | 4.14% |
|  |  |  |  | IGG | 923893 | 60365 | 6.53% |
|  |  |  |  | IGM | 117722 | 41973 | 35.65% |
| KD | CAL3 | 3926919 | 253088 | IGA | 550659 | 33823 | 6.14% |
|  |  |  |  | IGG | 1693028 | 62258 | 3.68% |
|  |  |  |  | IGM | 1683232 | 157006 | 9.33% |
| KD | CAL4 | 3132782 | 241951 | IGA | 1437270 | 29325 | 2.04% |
|  |  |  |  | IGG | 533313 | 28190 | 5.29% |
|  |  |  |  | IGM | 1162199 | 184435 | 15.87% |
